# A dual light-controlled co-culture system enables the regulation of population composition

**DOI:** 10.1016/j.synbio.2025.02.012

**Published:** 2025-02-19

**Authors:** Wei Jiang, Yijian Guo, Xuanshuo Liang, Ying Zhang, Jianning Kang, Zhengxin Jin, Bin Ning

**Affiliations:** a Central Hospital Affiliated to Shandong First Medical University, Jinan, 250013, Shandong, PR China; b West China Medical Center, Sichuan University, Chengdu, 610041, Sichuan, PR China; c Jinan Central Hospital, Shandong University, Jinan, 250013, Shandong, PR China; d Medical Integration and Practice Center, Shandong University, Jinan, 250013, Shandong, PR China

**Keywords:** Optogenetic switch, Co-culture system, Dual light-controlled system, Population ratio regulation

## Abstract

With the development of metabolic engineering, increasing requirements for efficient microbial biosynthesis call for establishment of multi-strain co-culture system. Dynamic regulation of population ratios is crucial for optimizing bioproduction performance. Optogenetic systems with high universality and flexibility have the potential to realize dynamic control of population proportion. In this study, we utilized an optimized chromatic acclimation sensor/regulator (CcaS/R) system and a blue light-activated YF1-FixJ-PhlF system as induction modules. A pair of orthogonal quorum sensing systems and a toxin-antitoxin system were employed as communication module and effector module, respectively. By integrating these modules, we developed a dual light-controlled co-culture system that enables dynamic regulation of population ratios. This co-culture system provides a universal toolkit for applications in metabolic engineering and synthetic biology.

## 1 Introduction

Advancements in synthetic biology and metabolic engineering have expanded the possibilities for cell-based engineered systems. As a prevalent method in metabolic engineering, co-culture systems address challenges that conventional methods often struggle to overcome [1]. In contrast to monoculture-based metabolic engineering, co-culture systems offer several significant advantages. First, co-culture systems alleviate the metabolic burden on each strain through division of labor [[2], [3], [4]]. Second, the incorporation of multiple strains fosters a diverse cellular milieu conductive to the functional expression of genes across various pathways. The biosynthetic pathway involves a multitude of enzymes with diverse biochemical properties; however, a monoculture system provides only a singular cellular context that may not accommodate the expression requirements of all genes [3,5]. Therefore, series of co-culture systems involving different strains have been established [[5], [6], [7], [8]]. Lastly, co-culture systems mitigate interference among different pathways or modules. Co-culture engineering supports plug-and-play biosynthesis of diverse target products, thereby avoiding the tedious work of optimizing entire metabolic pathways from scratch and enhancing the flexibility of individual modules [1].

Regulating population composition represents a major challenge in metabolic engineering co-culture technology. In numerous application scenarios, the population ratio is a critical determinant for optimizing production processes. Current strategies for managing population ratios primarily involve adjusting initial inoculation ratios and introducing necessary strains throughout the cultivation process [3,9]. However, these approaches do not enable dynamic control. In our previous research, we established a synthetic microbial consortium designed to achieve dynamic regulation of population ratios through the use of chemical inducers [10]. However, the efficacy of chemical inducers is constrained by their potential toxicity and high costs [[11], [12], [13]]. Additionally, the removal of residual inducers presents a significant challenge, particularly in research endeavors requiring precise temporal modulation of gene expression.

Optogenetics, a convergence of optics and genetic engineering, enables precise manipulation of diverse cellular activities [14]. In recent years, optogenetic technology has developed rapidly due to its advantages of non-invasiveness, high spatiotemporal specificity, rapid reversibility, and minimal toxicity [[15], [16], [17], [18], [19], [20], [21]]. Researchers have developed a variety of optogenetic tools that enable the control of cellular gene transcription, expression and editing by utilizing a spectrum of light wavelengths ranging from ultraviolet to far-red light [[22], [23], [24], [25], [26], [27], [28], [29]]. For instance, the pMag/nMag sensor responds to blue light [30], the Cph8/OpmR sensor is activated by red light [31], and the CcaS/R sensor is modulated by both green and red light [32]. The diversity of light sensors has inspired in-depth research into their engineering and applications. Optogenetic tools have been harnessed to modulate metabolic pathways and regulate specific protein activities [26,28,33,34]. Several optogenetic systems enabling the dynamic regulation of cell growth rates and co-culture composition have been established [35,36]. In these systems, only the growth rate of a single strain can be regulated.

Building on the advantages of optogenetic systems, we integrated two light control systems into the design of a dual light-controlled co-culture system, serving as the induction module. This system was designed and constructed based on the synthetic microbial consortia we previously developed, with the aimed of regulating population composition [10]. A toxin-antitoxin system was implemented as the effector module, while two orthogonal quorum sensing systems were incorporated to function as the communication module. Through characterization using multi-detection microplate reader, confocal microscopy and flow cytometer, we demonstrated that this system can effectively regulate population composition as anticipated. This co-culture system offers a versatile tool for synthetic biology and metabolic engineering.

## 2 Materials and methods

### 2.1 Strains, plasmids and culture media

All strains and plasmids used in this study are detailed in Table S1. The plasmid maps are shown in Figure S4 *E.* *coli* DH5α was used to construct plasmids, and *E. coli* TOP10 was utilized for characterization. Luria-Bertani (LB) medium, consisting of 5 g/L yeast extract, 10 g/L tryptone, and 10 g/L NaCl, served as the growth medium for both plasmid construction and strain characterization. To maintain plasmid stability and select for recombinants, antibiotics were added to the medium at the following concentrations: chloramphenicol (34 μg/mL), kanamycin (25 μg/mL), and ampicillin (100 μg/mL).

### 2.2 Characterization of the light-controlled quorum sensing system

The light-controlled quorum sensing system was characterized by assessing GFP fluorescence intensity across a spectrum of light wavelengths using a Multi-Detection Microplate Reader (Synergy HT, BioTek, Winooski, VT, USA). Single colonies were cultivated in test tubes containing 5 mL of LB medium, supplemented with appropriate antibiotics, at 37 °C for approximately 12 h. The precultures were then transferred to a 12-well microassay plate containing 2 mL of LB medium at a 2 % (v/v) inoculation ratio and incubated at 37 °C. The LED beads used in this study have specific wavelength ranges (Shenzhen Hongji Core Electronics): 520–525 nm (green light), 620–625 nm (red light), and 460–463 nm (blue light). The cultures were then exposed to green (1.96 W/m^2^), red (3.92 W/m^2^), and blue light (8.66 W/m^2^) conditions, as well as darkness. GFP fluorescence was quantified using an excitation wavelength of 485 nm and an emission wavelength of 528 nm, normalized to the optical density at 600 nm (OD_600_).

### 2.3 Characterization of the light-controlled system via multi-detection microplate reader

Strains were inoculated in LB liquid medium containing the corresponding antibiotics and cultured overnight at 37 °C. Then, two strains were transferred to a 12-well plate at a 2 % inoculation ratio, maintaining a 1:1 ratio. Following an overnight incubation under red light irradiation, the cultures were subsequently exposed to green or blue light for the specified duration. GFP and RFP fluorescence levels were then quantified using Multi-Detection Microplate Reader at intervals. GFP fluorescence was quantified with an excitation wavelength of 485 nm and an emission wavelength of 528 nm, RFP fluorescence was measured using an excitation wavelength of 590 nm and an emission wavelength of 645 nm.

### 2.4 Characterization of the light-controlled system through confocal microscope

A confocal laser scanning microscope (Leica TCS SP8) was used to visualize the composition of two strains in microbial co-cultures. The strains were similarly inoculated and incubated in LB liquid medium with the appropriate antibiotics under the same conditions as described above. After overnight incubation under red light, the cultures were exposed to either green or blue light for the required duration. Samples were prepared by washing with phosphate-buffered saline (PBS) and 5 μL samples were imaged. The excitation wavelengths were 488 nm for green fluorescence and 552 nm for red fluorescence, with emission detection ranges of 500–540 nm and 560–640 nm, respectively. The microscope was operated at 20 × magnification.

### 2.5 Characterization of the light-controlled system by flow cytometer

The methods of strain culture and light induction were the same as above. Prior to flow cytometry analysis, strains were washed and diluted to OD less than 0.1 with PBS. Forward scatter and side scatter were used to determine microflora. A total of 20,000 events were collected for each sample. Green fluorescence and red fluorescence were detected in FITC channel and PE-Texas Red channel, respectively.

## 3 Results

### 3.1 Design and construction of the dual light-controlled co-culture system

To establish the light-controlled co-culture system, we employed three distinct modules, each performing specific functions. Two optogenetic systems that we had previously optimized were utilized as the induction module [37]; two orthogonal quorum sensing systems that we had developed served as the communication module [38]; and the toxin-antitoxin system (CcdA/B) was chosen as the effector module.

Concurrent operation of the two optogenetic systems requires the orthogonality between them. Interference among regulatory systems can result in unexpected behaviors and cause the loss of circuit functions [39,40]. Accordingly, we selected two optogenetic systems that we had previously verified their orthogonal relationship [37]. One of these is the refined CcaS/R system, comprising the photoreceptive protein CcaS and the response regulator CcaR [41,42]. Upon exposure to 535 nm green light, the CcaS photoreceptor undergoes autophosphorylation, triggering a cascade that transfers phosphate to CcaR, thereby activating gene transcription driven by the P_cpcG2_ promoter. Conversely, this process is reversed under 670 nm red light [43]. Additionally, the expression of the heterologous genes heme oxygenase1 (*ho1*) and ferredoxin oxidoreductase (*pcyA*) facilitates the synthesis of phycocyanobilin, which is essential for enhancing the CcaS photoreceptor's response [43]. In previous study, a PAS domain-shortened CcaS#10 variant was constructed to broaden response ranges and reduce expression leakage [23,24]. Building on this, we established an optimized CcaS/R system (RBS10-CcaS#10-CcaR) with an increased dynamic range in our preceding research [37], and this optimized CcaS/R system was utilized in this study. Additionally, we selected the YF1-FixJ system as another optogenetic module. The native YF1-FixJ is activated under dark conditions, with its activity suppressed upon blue light exposure [44]. A blue light-activated YF1-FixJ-PhlF system was engineered by integrating the repressor protein PhlF [33]. The transcription of PhlF is controlled by the blue light-sensitive promoter P_FixK2_ and the target gene positioned downstream of the PhlF-binding operator. Under dark conditions, PhlF expression is induced and repressed the target gene's expression. In contrast, blue light facilitates the transcription of the target gene (Fig. S1).

We deployed two completely orthogonal quorum sensing systems, the tra∗ system and the las system, as the communication module [38]. The tra∗ system, an optimized version of the tra system, comprises the regulator TraR (W), the promoter P_tra∗_, and the HSL synthase EsaI, which produces 3OC6HSL. The las system includes the regulator LasR, the promoter P_las_, and the HSL synthase LasI, which produces 3OC12HSL. These systems enable non-interference communication between the strains. For the effector module, we selected a *ccd* operon-encoded toxin-antitoxin system, which includes two genes: *ccdA* and *ccdB*. The toxic protein CcdB inhibits DNA gyrase, leading to cell death, whereas the antitoxic protein CcdA protects the cell from the lethal effects of CcdB [45].

**Fig. 1:**
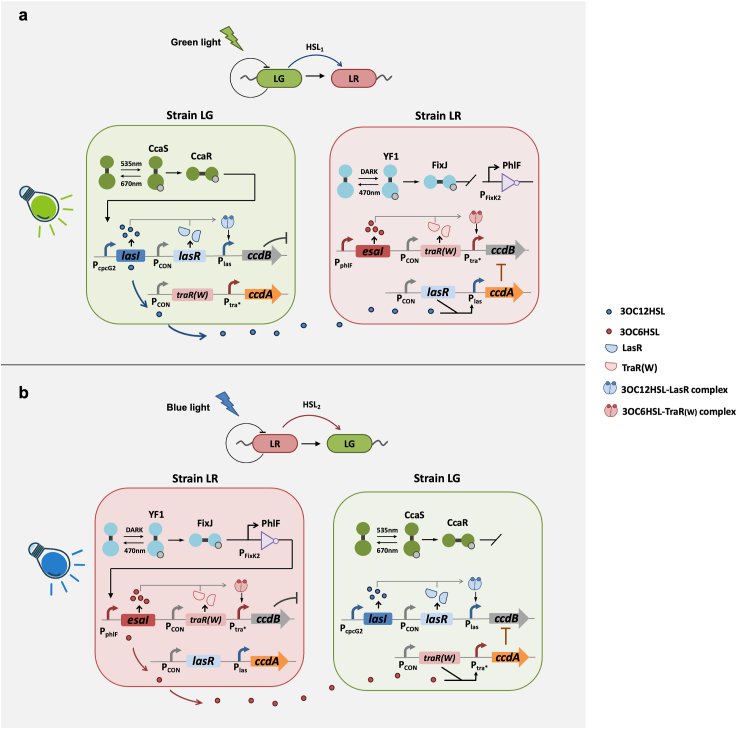
Schematic representation of the dual light-controlled co-culture system, illustrating the interaction mechanisms under green light (a) and blue light (b). When exposed to green light, strain LG produces 3OC12HSL and diffuses into strain LR, triggering the expression of CcdB in strain LG and CcdA in LR. When exposed to blue light, strain LR produces 3OC6HSL and diffuses into strain LG, triggering the expression of CcdB in strain LR and CcdA in LG.

Two strains, LR and LG, were engineered in the co-culture system (Fig. 1). The genomes of strains LR and LG were integrated with red fluorescent protein (RFP) and green fluorescent protein (GFP), respectively. In strain LR, the YF1-FixJ-PhlF system was designed to induce the tra∗ system, thereby regulating the transcription of *ccdB*. Additionally, the LasR receptor protein of the las system and the antitoxin protein CcdA were co-expressed in strain LR. In strain LG, the CcaS/R system was engineered to activate the las system, which in turn governs the transcriptional activity of *ccdB*. The receiver protein of the tra∗ system, TraR, along with the antitoxin protein CcdA, was also expressed in strain LG.

When exposed to green light, strain LG produces 3OC12HSL (*N*-(3-oxododecanoyl)-homoserine lactone) signal molecules via the HSL synthase LasI in the las system, which can also diffuse into strain LR. When sufficient signals accumulated, they bind to the receptor LasR, triggering the expression of CcdB in strain LG and CcdA in LR. Under this condition, strain LG initiates a negative feedback loop via the *cis*-acting HSL activation of toxin expression, which leads to self-limitation. Simultaneously, positive feedback is established, conferring protection to strain LR through the *trans*-acting HSL activation of antitoxin expression. Consequently, strain LR becomes dominant within the co-culture system upon green light exposure (Fig. 1a). Conversely, when irradiated with blue light, strain LR generates 3OC6HSL (*N*-(3-oxohexanoyl)-homoserine lactone) signal molecules through the HSL synthase EsaI in the tra∗ system, which can also diffuse into strain LG. Upon reaching a threshold concentration, 3OC6HSL bind to the receptor TraR, activating CcdB in strain LR and CcdA in strain LG. In this scenario, strain LR establishes a negative feedback loop via the *cis*-acting HSL activation of toxin expression, which results in self-limitation. Simultaneously, a positive feedback loop is formed, providing protection to strain LG via the *trans*-acting HSL activation of antitoxin expression. As a result, strain LG assumed dominance within the co-culture system when exposed to blue light (Fig. 1b).

The optical control hardware we employed consisted of a custom-designed LED light board (Fig. S2). We selected LED beads with specific wavelength ranges: 520–525 nm (green light) and 620–625 nm (red light) for the dual-color lamp beads, and 460–463 nm for the blue lamp beads. These lamp beads were mounted on a breadboard aligned with the well positions of a 12-well microassay plate. A 12 V/36 mA ballast was integrated into the circuit to convert the standard alternating current from the power supply into the direct current required for LED operation. The light intensities for the green, blue and red light were 1.96 W/m^2^, 8.66 W/m^2^ and 3.92 W/m^2^, respectively.

### 3.2 Characterization of individual strains within the dual light-controlled co-culture system

**Fig. 2:**
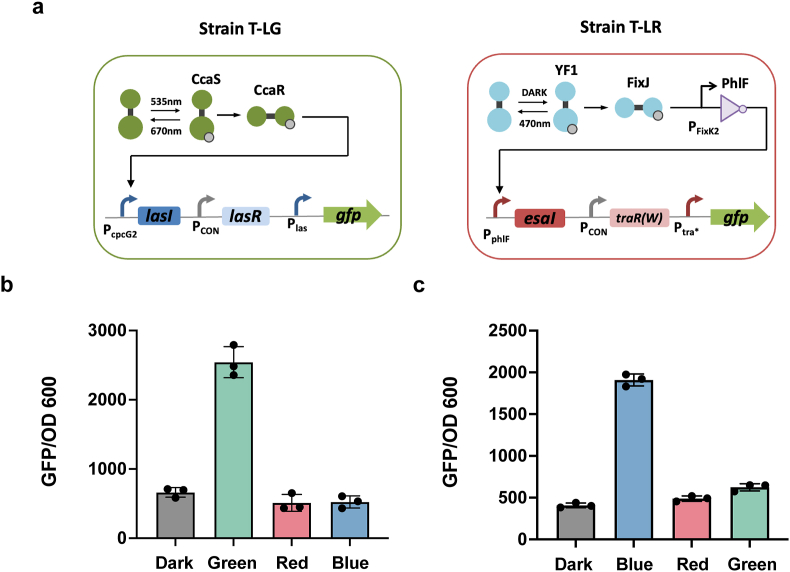
Characterization of the light-responsive quorum sensing system. (a) Schematic depiction of the genetically engineered strains T-LG and T-LR designed to evaluate light-controlled quorum sensing. (b) Characterization of strain T-LG under different light conditions: green, red, blue, and darkness. (c) Characterization of strain T-LR under different light conditions: green, red, blue, and darkness. Data were detected by using Multi-Detection Microplate Reader. Data show the means ± SD of three experiments.

We first evaluated the light-modulated quorum sensing system to determine the efficacy of optogenetic induction. To this end, we designed and constructed two verification strains, T-LG and T-LR, with GFP as the reporter for both strains (Fig. 2a). In strain T-LG, the CcaS/R system activates the las system; while in strain T-LR, the YF1-FixJ-PhlF system activates the tra∗ system. Strains T-LG and T-LR were characterized under various conditions, including green, red, and blue light, as well as darkness, to assess their responses (Fig. 2b and c). Strain T-LG exhibited significant activation under green light irradiation (Fig. 2b). Strain T-LR showed activation under blue light, with a weak crosstalk activation observed under green light (Fig. 2c). We speculated that this level of crosstalk will not breakdown the functionality of the co-culture system. To standardize the off-state for the co-culture system, it was necessary to establish a uniform light control mode that would serve as the off-state for both optogenetic systems. Our observations revealed that the mean GFP expression in strain T-LG was higher under dark conditions than under red light exposure (Fig. 2b). This finding suggests a subtle activating influence of darkness on the CcaS/R system, consistent with the findings reported by Tabor et al. [24]. Consequently, red light irradiation was selected to uniformly represent the off-state for both light systems.

**Fig. 3:**
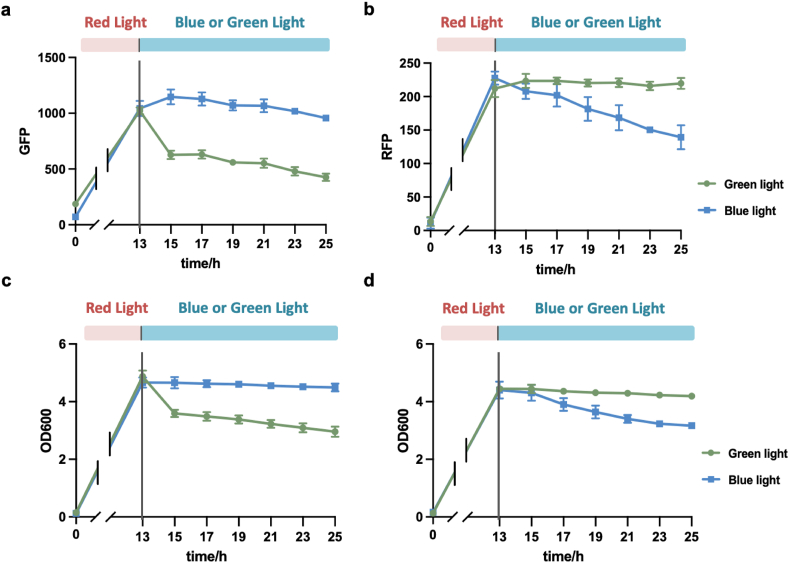
Characterization of each strain within the dual light-controlled co-culture system. (a) Fluorescence characterization of strain LG. (b) Fluorescence characterization of strain LR. (c) Growth profile of strain LG. (d) Growth profile of strain LR. Each strain was exposed to red light overnight, followed by a 12-h exposure to either blue or green light. Fluorescence data were detected by using Multi-Detection Microplate Reader. OD_600_ was detected by using spectrophotometer. Data show the means ± SD of three experiments.

The co-culture system was engineered to incorporate two strains, LG and LR, according to the design described above. The accumulation of dead bacteria interfered with the detection of cell density, affecting the OD_600_ values [19]. Additionally, it was difficult to distinguish between different strains using OD_600_ in the co-culture systems. Given this potential limitation of cell density detection, we employed both OD_600_ and fluorescence measurements to validate the system's functionality. We first characterized each strain under green and blue light conditions (Fig. 3). Strains were cultured overnight under red light, followed by 12 h of exposure to either green or blue light. As expected, strain LG exhibited significant inhibition under green light irradiation. This observation confirms the successful activation of the las system by the CcaS/R system, leading to the expression of CcdB (Fig. 3a). Strain LR was inhibited under blue light, demonstrating that the YF1-FixJ-PhlF system can regulate the expression of CcdB via the tra∗ system. A similar trend was observed in cell growth (Fig. 3c and d). These findings indicate that each strain within the dual light-controlled co-culture system can modulate its growth in response to specific light wavelengths.

### 3.3 Characterization of the dual light-controlled co-culture system

**Fig. 4:**
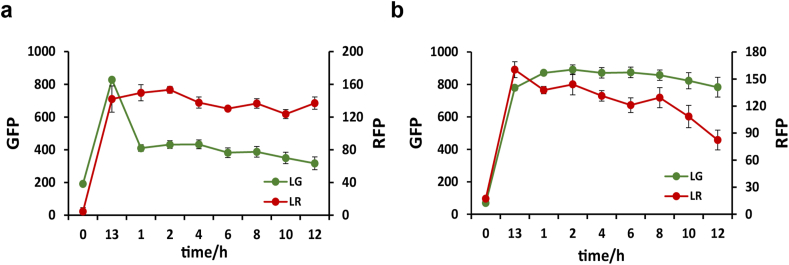
Fluorescence characterization of the dual light-controlled co-culture system under green light (a) and blue light (b). Co-culture system was exposed to red light overnight, followed by either blue or green light for 12 h. Fluorescence data were detected by using Multi-Detection Microplate Reader. Data show the means ± SD of three experiments.

We characterized the dual light-controlled co-culture system using equal inoculation (1:1) of strains LG and LR. The strains were initially co-cultured under red light overnight. Following this, the co-culture system was exposed to blue or green light. Upon exposure to green light, the growth of strain LG was markedly suppressed, while the growth of strain LR remained unaffected (Fig. 4a). Conversely, when the co-culture system was radiated with blue light, the growth of strain LR was suppressed (Fig. 4b). These results confirm that the co-culture system we designed is capable of regulating population composition as intended.

**Fig. 5:**
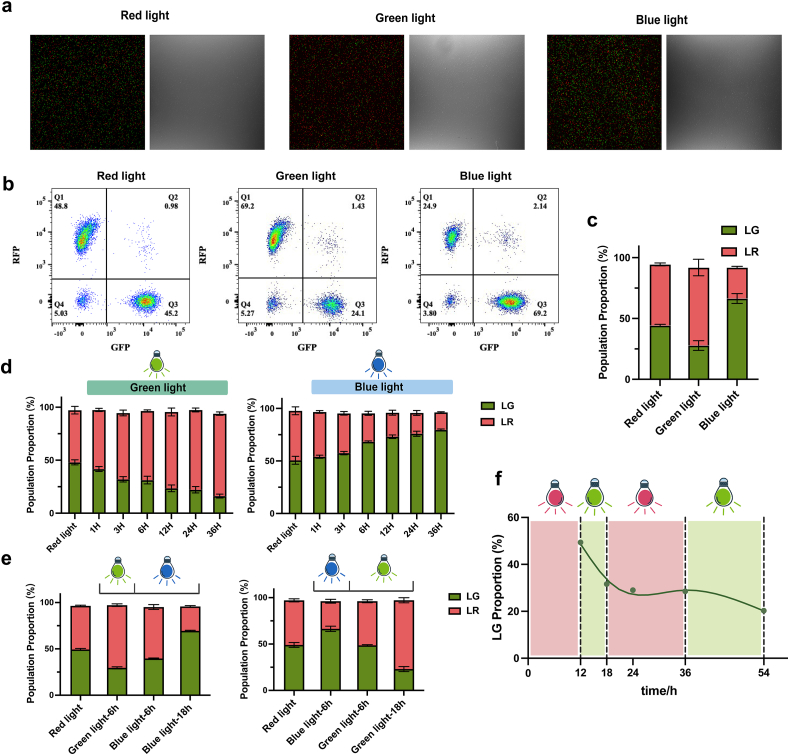
Characterization of the dual light-controlled co-culture system. (a) Confocal microscopy analysis of the co-culture system following overnight red light induction and subsequent 12-h blue/green light induction. The left side of each set of images is the fluorescent image, and the right side is the bright field image. (b) Population composition assessed by flow cytometry following overnight red light induction and subsequent 12-h blue/green light induction. Events in quadrant Q1 represent strain LR. Events in quadrant Q3 represent strain LG. Events in quadrant Q2 are cell aggregates. Events in quadrant Q4 are impurities and dead cells. Data are from one representative sample of three independent biological replicates. All replicates are shown in Fig. S3. (c) Population proportion analysis of LG and LR for the characterization results in (b). (d) Investigation of the temporal dynamics of population proportions for 36 h. (e) Dynamic regulation characterization by alternating the light conditions between blue and green. (f) Characterization of the reversibility by altering the light conditions. Data show the means ± SD of three experiments.

To access changes in the proportion of cells more accurately and intuitively, we used confocal microscopy (Fig. 5a) and flow cytometer (Fig. 5b and c) to monitor alterations in population composition. Strains were inoculated in an equimolar ratio and co-cultured overnight under red light irradiation, after which the population composition was examined. The co-culture system was then exposed to green and blue light for 12 h in separate experiments. After overnight co-cultivation under red light, the ratio of strains LG to LR remained approximately at the initial 1:1 equilibrium, with the proportions of strains LG and LR being 45.2 % and 48.8 %, respectively (Fig. 5a, b, c; Fig. S3). Following 12 h of green light exposure, there was a significant reduction in the proportion of strain LG, with the ratio decreasing to 24.1 %, while the ratio of strain LR increased to 69.2 % (Fig. 5b, c; Fig. S3). Conversely, after 12 h of blue light irradiation, the proportion of strain LR decreased significantly, resulting in a ratio of 24.9 % for strain LR and 69.2 % for strain LG (Fig. 5b, c; Fig. S3). We further investigated the changes in system composition over time (Fig. 5d). The results demonstrated that the strain ratios began to change as early as 3 h. Additionally, we observed that prolonged light exposure (36 h) led to continued changes in population proportions. This phenomenon may result from the continuous expression of the toxin protein in the light-activated strain, causing a sustained growth disadvantage.

To further demonstrate the system's capability for dynamic regulation, we performed characterization by alternating the light conditions between blue and green (Fig. 5e). The results indicate that when the light shifts from green to blue, the dominant strain shifts from LR to LG; conversely, when the light shifts from blue to green, the dominant strain shifts from LG to LR (Fig. 5e). These findings demonstrate the system's ability to dynamically regulate population ratios.

Since one of the main advantages of light-induction systems over other systems, such as chemical inducers, is their reversibility, we further investigated whether this feature could be achieved by our system (Fig. 5f). After 12 h of cultivation under red light with equal inoculation, the system was exposed to green light for 6 h (from 12 to 18 h) to induce activation. During this period, the proportion of strain LG decreased. Subsequently, the light was switched to red (from 18 to 36 h) to examine whether the system's activated state could be terminated by altering the light conditions. During this period, we observed that the strain ratio did not exhibit significant changes. The system was reactivated by reapplying green light (from 36 to 54 h), and the ratio of strain LG declined a second time. These results demonstrate that our system can transition between activated and deactivated states by altering light conditions, thereby confirming the reversibility of the light-controlled induction system. This feature provides a significant advantage over chemical inducers, which are often difficult to remove, offering greater flexibility in regulation and controllability.

These results demonstrate that the composition of strains can be regulated through light variations, confirming the efficacy of the dual light-controlled co-culture system we developed. Meanwhile, this system harnesses the unique advantages of the optogenetic switch, allowing dynamic and reversible regulation of population composition. However, it is often necessary to maintain a stable ratio between populations in various production applications, motivating us to further investigate the system's ability to sustain stable population ratios.

### 3.4 Stable population proportionality attained under concurrent dual-light induction

**Fig. 6:**
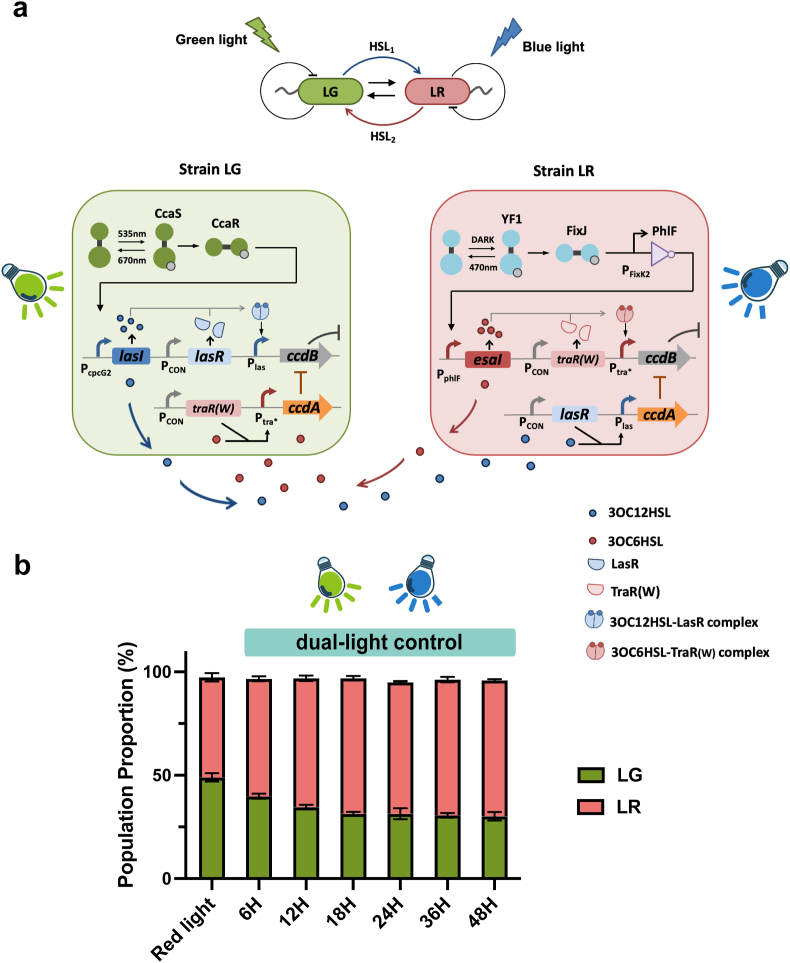
Stable population proportionality attained under concurrent dual-Light induction. (a) Schematic representation of the dual light-controlled co-culture system when exposed to simultaneous two-light illumination. (b) Characterization of the dual light-controlled co-culture system when exposed to simultaneous two-light illumination for 48 h. Data show the means ± SD of three experiments.

The design of this system relies on the interactions between the populations. The populations form a symbiotic ecological relationship when exposed to simultaneous two-light illumination (Fig. 6a). Specifically, strain LG produces 3OC12HSL under green light, activating CcdB expression in LG and CcdA expression in strain SR. Meanwhile, strain SR produces 3OC6HSL under red light, leading to CcdB expression in strain SR and CcdA expression in strain LG. Consequently, the two strains exhibit mutually protective behaviors, establishing a symbiotic relationship between LG and LR. Symbiotic relationships have the potential to maintain system stability. Thus, we characterized the population composition by inducing with green and blue light simultaneously. We found that this symbiotic interaction promotes stable population ratio regulation (Fig. 6b). The results demonstrate that when both lights are applied simultaneously, the population ratio stabilizes after 12 h and remains stable for up to 48 h. This finding demonstrates the system's ability to maintain strain proportions through the strategic design of symbiotic interactions between the two strains. Stable regulation of population ratios is a key feature that supports the broader applications in metabolic engineering. We anticipate that modulating light intensity in future studies could allow the system to achieve a broad range of strain ratios. Establishing this model provides a foundation for the application of co-culture systems.

## 4 Discussion

In this study, we designed and established a dual light-controlled co-culture system by integrating three key modules: an induction module, a communication module, and an effector module. This system effectively regulates population composition. The optogenetic switch, functioning as the induction module, allows for non-invasive, highly temporally specific, and low-toxicity manipulation of population composition, making it highly applicable in biotechnology fields such as metabolic engineering.

In our system, when single-light induction was used, population composition continued to change over time. This characteristic suggests potential applications in co-culture production processes. Specifically, this system is applicable in scenarios where a dominant strain is required in the later stages of co-culture to allocate resources for product synthesis. For instance, the product-producing strain could dominate in later stages to reduce resource competition with substrate-degrading strains. When both lights were applied concurrently, the population composition would reach a steady state. This characteristic suggests another potential application of this stable system in co-culture production processes. Specifically, when the synthesis pathway is divided between two strains requiring labor division for product production, maintaining a stable co-culture state is essential.

The orthogonal quorum sensing systems incorporated into this design serve two critical functions: 1) ensuring non-interfering intercellular communication, which facilitates the establishment of interactive relationships within the consortium. Specifically, the expression of antitoxin in each strain is induced by signals produced by the other strain, resulting in mutually protective behaviors. This interaction enhances the system's stability in potential application scenarios. 2) providing a buffering mechanism for cell growth. In previous studies, we established a control system that directly induced the toxin-antitoxin system without using QS systems, in which the growth of strains was significant inhibited [10]. Therefore, the QS system's growth buffering function is essential for maintaining strain viability and provides a robust foundation for population regulation. Moreover, this dual light-controlled co-culture system does not rely on a specific metabolic pathway, offering a versatile toolkit for various applications in metabolic regulation.

Further refinement and characterization of this system are necessary, such as modulating light intensity and diversifying light strength combinations to facilitate precise and variable control over the population ratios. Additionally, incorporating advanced hardware will be crucial for the nuanced calibration of the system's parameters.

## 5 Conclusion

In this research, we established a dual light-controlled co-culture system capable of modulating population composition dynamically through the use of distinct light wavelengths. This system enables population regulation in a non-invasive, highly temporally specific, and reversible manner. Additionally, stable population proportionality can be attained under concurrent dual-Light induction. The dual light-controlled co-culture system offers an innovative and versatile toolkit for applications in metabolic engineering and synthetic biology. Establishing of this model provides direction for further optimization to meet the needs of practical applications.

## CRediT authorship contribution statement

**Wei Jiang:** Writing – original draft, Visualization, Validation, Methodology, Funding acquisition, Data curation, Conceptualization, Writing – review & editing. **Yijian Guo:** Conceptualization, Data curation, Formal analysis, Investigation, Validation, Visualization, Writing – review & editing. **Xuanshuo Liang:** Visualization, Resources, Data curation, Formal analysis, Software, Writing – review & editing. **Ying Zhang:** Writing – review & editing, Validation. **Jianning Kang:** Validation, Resources. **Zhengxin Jin:** Formal analysis, Data curation. **Bin Ning:** Supervision, Resources, Project administration, Methodology, Funding acquisition.

## Declaration of competing interest

The authors declare that they have no known competing financial interests or personal relationships that could have appeared to influence the work reported in this paper.
