# Supplementary material for: A dual light-controlled co-culture system enables the regulation of population composition
Source: Synth Syst Biotechnol. 2025 Feb 19;10(2):574–82. doi: 10.1016/j.synbio.2025.02.012 (PMC11910626; doi:10.1016/j.synbio.2025.02.012)
Supplement: Multimedia component 1 [file mmc1.docx]

Supporting Information

**A Dual Light-controlled Co-culture System enables the Regulation of** **Population Composition**

Wei Jiang^a, d#^, Yijian Guo^c#^, Xuanshuo Liang^b^, Ying Zhang^a^, Jianning Kang^c^, Zhengxin Jin^c^, Bin Ning^a,c^*

^a^ Central Hospital Affiliated to Shandong First Medical University, Jinan, 250013, Shandong, PR China

^b^ West China Medical Center, Sichuan University, Chengdu, 610041, Sichuan, PR China

^c^ Jinan Central Hospital, Shandong University, Jinan, 250013, Shandong, PR China

^d^ Medical Integration and Practice Center, Shandong University, Jinan, 250013, Shandong, PR China

^#^ Wei Jiang and Yijian Guo contributed equally to this work

***Corresponding author:** Bin Ning

E-mail: bning@sdfmu.edu.cn

**
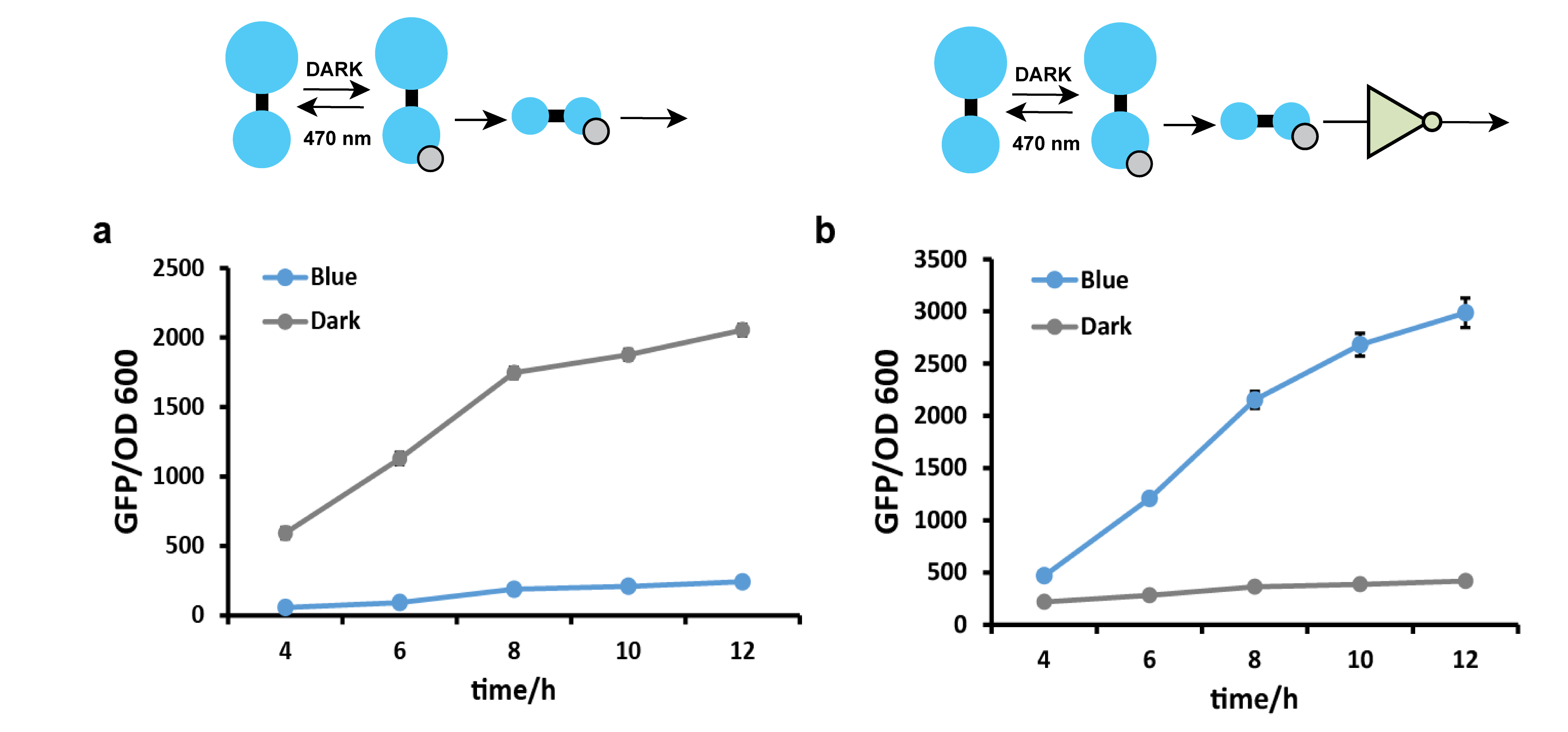
**

**Figure S1.** Characterization of YF1-FixJ optogenetic systems. (a) Characterization of native YF1-FixJ optogenetic system. (b) Characterization of YF1-FixJ-PhlF optogenetic system.


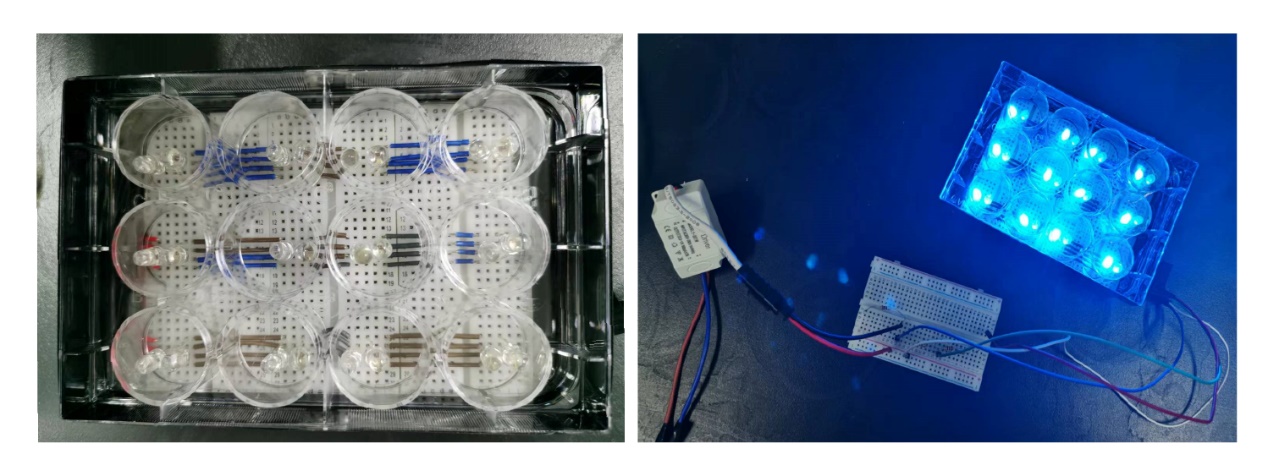


**Figure S2.** Hardware equipment of light-controlled characterization in 12-well microassay plate.

**
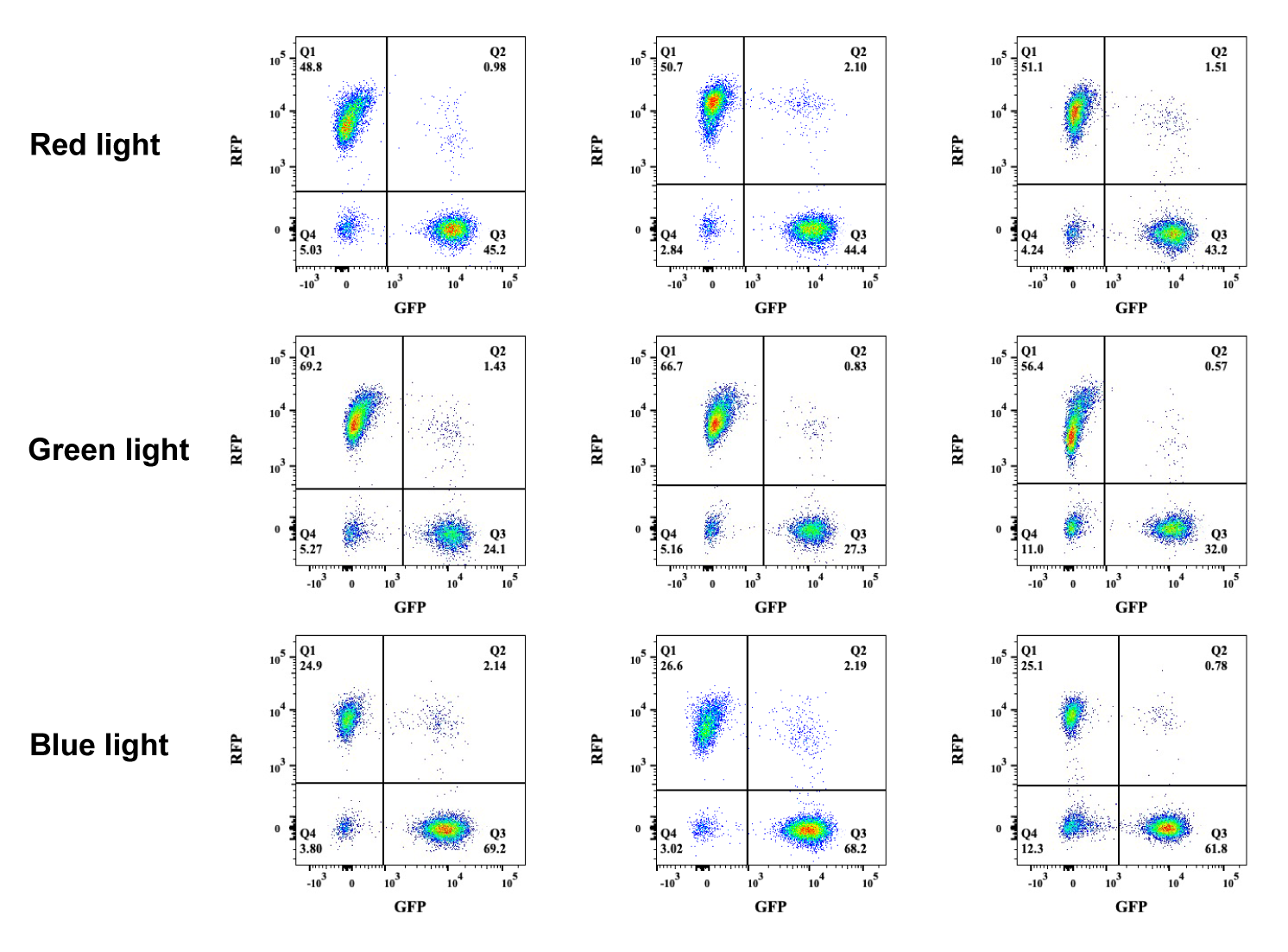
**

**Figure S3.** Characterization statistics of light-controlled bacterial co-culture system by confocal microscopy. (a) Characterization statistics of co-culture system after overnight exposure to red light. (b) Characterization statistics of co-culture system after 12 hours of green light irradiation. (c) Characterization statistics of co-culture system after 12 hours of blue light irradiation.

**
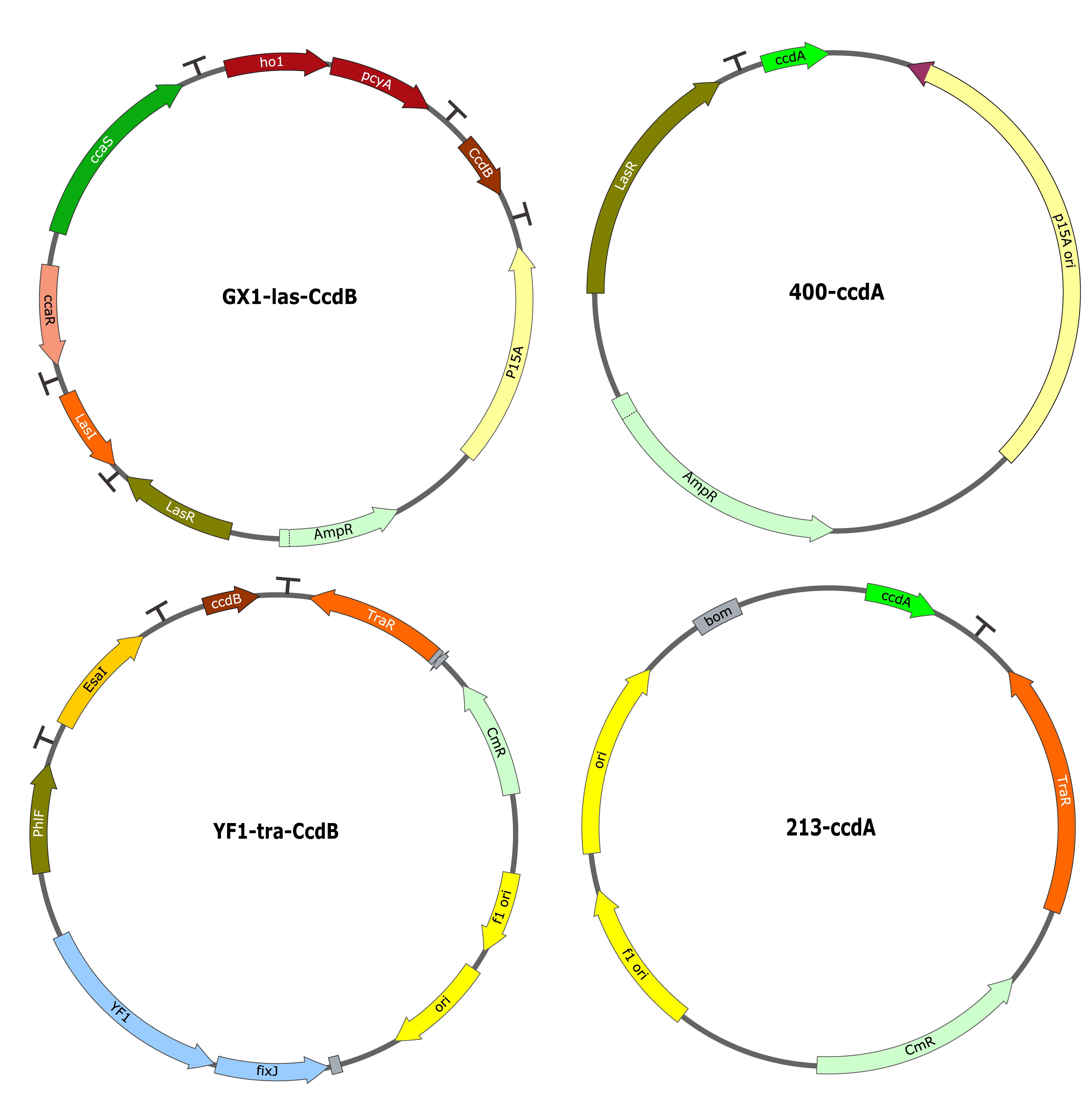
**

**Figure S4.** Plasmid maps of key plasmids constructed and used in this study.

**Table S1. Strains and plasmids used in this study**

| **Strains and plasmids** | **Relevant properties** | **Source** |
| --- | --- | --- |
| **Strains** |  |  |
| T-LG | TOP10 carrying GX1-las | This study |
| T-LR | TOP10 carrying YF1-tra | This study |
| TOP10-GFP | GFP is integrated into the TOP10 genome at *attp* | Lab Stock |
| TOP10-RFP | RFP is integrated into the TOP10 genome at *attp* | Lab Stock |
| LG | TOP10-GFP carrying 213-ccdA and GX1-las-CcdB | This study |
| LR | TOP10-RFP carrying 400-ccdA and YF1-tra-CcdB | This study |
| **Plasmids** |  |  |
| GX1-las | P15A ori, Amp^R^, *ho1*, *pcyA*, CcaS, CcaR, *cpcG2* promoter, *lasI*, *lasR*, *las* promoter, GFP | This study |
| YF1-tra | ColE1 ori, Cm^R^, YF1, FixJ, PhIF, *easI*, *traR*, *tra**promoter, GFP | This study |
| 400-ccdA | P15A ori, Amp^R^, *lasR*, *tra**promoter, *ccdA* | Lab Stock |
| 213-ccdA | ColE1 ori, Cm^R^, *traR*, *las* promoter, *ccdA* | Lab Stock |
| GX1-las-CcdB | P15A ori, Amp^R^, *ho1*, *pcyA*, CcaS, CcaR, *cpcG2* promoter, *lasI*, *lasR*, *las* promoter, CcdB | This study |
| YF1-tra-CcdB | ColE1 ori, Cm^R^, YF1, FixJ, PhIF, *easI*, *traR*, *tra**promoter, CcdB | This study |

**Table S2. Sequences of key genes used in this study**

| Gene | Sequence |
| --- | --- |
| ccaS | atgggcaaatttctaattccaatcgaatttgtttttctggcgatcgccatgacctgttatttatggcacagacaaaaccaagaacgccgcaggattgaaattagcatcaagcaacaaacccaacgggaacgatttattaaccaaattacccaacatatccgccaatctttaaacttggaaacggttttaaataccaccgtcgctgaagttaaaaccctgttgcaagttgatcgagttctaatttatcgcatttggcaagatggcacgggcagcgccattacggaatcggtgaatgccaattatcctagtattttagggcggaccttttccgatgaagtttttcccgttgaataccatcaagcctacaccaaaggtaaagtacgggccattaatgacattgaccaggatgacatagagatttgcctagctgatttcgtcaaacaatttggcgtgaaatcaaaattagtagtgcccattcttcaacataatcgtgcttcttccctagataatgaatcagaatttccctatctttgggggctgttaattacccatcaatgtgcttttacccggccatggcaaccgtgggaagtggagttaatgaaacagctagccaatcaggtcgcgatcgccatccaacaatcggaattatatgagcaattacagctagctttagaacgggaaaaagaattaagccgcctaaaaactcgttttttctccatggcttcccatgaatttcgtactcccctcagtacggccttagctgctgcccaattactggaaaattctgaagtggcctggcttgatcccgataagcgtagccggaacttacaccgtattcaaaattccgtgaaaaatatggtacagctcctggatgatattttaatcattaaccgtgccgaagcgggcaaattggaatttaatcctaattggttagatttgaaattattgttccagcaatttatcgaagaaattcaattaagtgtcagtgaccaatattattttgactttatttgtagcgctcaagatacgaaggcattggtggatgaaaggttagtgcggtctattttatctaatctgttatctaatgcgattaaatactctcccgggggagggcagattaaaattgccctaagcctagattcggaacagattatttttgaagtcaccgaccagggcattggcatttcgccagaggaccaaaagcaaatttttgaaccctttcatcggggcaaaaatgtcagaaatattacgggaacaggactcggtttaatggttgccaagaaatgtgttgacttacacagtggcagtatcttgctaaaaagtgcagttgaccagggaacaacagttactatctgtttaaaacgctataaccatttgcctcgagcttag |
| ccsR | atgagaattcttttagtggaggatgatttgccgctggcggaaacccttgctgaagcattgagtgaccagctttacaccgttgatattgccaccgacgcttccctcgcctgggactatgcctcccgactggaatatgacctcgttattttggatgtgatgctgccggagttggacgggattaccctctgtcaaaaatggcgatcgcacagttatttaatgccaattttgatgatgacagccagggatacgatcaatgataaaatcacgggcttggatgcgggggcggatgattatgtggtcaagccagtggatttgggggagttatttgccagggtgcgagctttgttgcgtcggggttgtgcaacgtgccaaccagttttagagtgggggccaatcaggttggatccaagcacctatgaagttagttatgacaatgaggttttgtctttgacccgcaaggaatacagcattctggaattactactccgcaatggccgtcgggtgctaagtcggagcatgattatcgatagtatctggaagttggagagtcccccagaggaagatacggttaaggtgcatgtgcggagtttgcgacaaaaattaaaaagtgccggtttatcagcagatgccattgaaacggtccatggcattgggtatcgtctggccaatttaacggaaaaatctttgtgccaagggaaaaactag |
| lasR | atggccttggttgacggttttcttgagctggaacgctcaagtggaaaattggagtggagcgccatcctgcagaagatggcgagcgaccttggattctcgaagatcctgttcggcctgttgcctaaggacagccaggactacgagaacgccttcatcgtcggcaactacccggccgcctggcgcgagcattacgaccgggctggctacgcgcgggtcgacccgacggtcagtcactgtacccagagcgtactgccgattttctgggaaccgtccatctaccagacgcgaaagcagcacgagttcttcgaggaagcctcggccgccggcctggtgtatgggctgaccatgcCgctgcatggtgctcgcggcgaactcggcgcgctgagcctcagcgtggaagcggaaaaccgggccgaggccaaccgtttcatggagtcggtcctgccgaccctgtggatgctcaaggactacgcactgcagagcggtgccggactggccttcgaacatccggtcagcaaaccggtggttctgaccagccgggagaaggaagtgttgcagtggtgcgccatcggcaagaccagttgggagatatcggttatctgcaactgctcggaagccaatgtgaacttccatatgggaaatattcggcggaagttcggtgtgacctcccgccgcgtagcggccattatggccgttaatttgggtcttattactctcTGAtcttgcctctcaggtcggcgagctggcgatcggtaatttgcccttctatatagaaatgcaaaagcagatatatagggaagggcaggttctcgccattctcgaaacgactg |
| lasI | atgatcgtacaaattggtcggcgcgaagagttcgataaaaaactgctgggcgagatgcacaagttgcgtgctcaagtgttcaaggagcgcaaaggctgggacgttagtgtcatcgacgagatggaaatcgatggttatgacgcactcagtccttattacatgttgatccaggaagatactcctgaagcccaggttttcggttgctggcgaattctcgataccactggcccctacatgctgaagaacaccttcccggagcttctCcacggcaaggaagcgccttgctcgccgcacatctgggaactcagccgtttcgccatcaactctggacagaaaggctcgctgggcttttccgactgtacgctggaggcgatgcgcgcgctggcccgctaTagcctgcagaacgacatccagacgctggtgacggtaaccaccgtaggcgtggagaagatgatgatccgtgccggcctggacgtatcgcgcttcggtccgcacctgaagatcggcatcgagcgcgcggtggccttgcgcatcgaactcaatgccaagacccagatcgcgctttacgggggagtgctggtggaacagcgactggcggtttcatga |
| traR(W) | atgcagcactggctggacaagctgactgatcttgccgcgatcgaaggcgatgagtgcatcctgaagaccgggctggcggacatcgccgaccatttcggcttcaccggctatgcctaccttcatatccagcacaggcacatcaccgccgttaccaactatcaccgccaatggcaatcaacctacttcgacaagaagttcgaagcgctcgatccggtcgtcaaacgcgcgaggtcccggaagcacatcttcacctggtcgggcgagcacgagcggccgacgctgtcgaaggacgagcgtgccttctatgaccacgcatccgatttcggcatccgctccggcatcacaatacccatcaagaccgccaacggctttatgtcgatgttcacgatggcatcggacaagccggtgatcgatctcgatcgggagatcgatgcagtcgcagccgctgcaaccatcgggcagatccatgcccgcatctcattccttcgcaccacccctaccgcggaagatgccgcatggctcgatccgaaggaggccacctatctgagatggattgccgtcggcaagacgatgTGggagatcgccgacgtcgaaggggtcaagtacaacagcgtccgcgtcaagctacgcgaagccatgaagcgcttcgacgtccgcagcaaggcccatcttaccgcgctcgccatccggcggaaactcatctgagagttcgaagttcaagtttcgaaactgcgatccgggaggtctgctagtgtcaa |
| esaI | atgctggagctgttcgacgttagctatgaagaactgcagaccacccgtagcgaagagctgtacaaactgcgcaagaaaaccttctctgaccgcctgggttgggaagttatttgctctcagggcatggaatccgacgaatttgacggtccgggcacccgctacatcctgggtatctgcgaaggccagctggtatgtagcgttcgcttcacctctctggatcgtccgaatatgatcacccacacctttcagcattgtttcagcgatgtcactctgccagcctacggcaccgaatcctctcgtttcttcgtggacaaagcgcgtgcacgcgccctgctgggtgaacactacccgatcagccaggtactgttcctggcgatggtgaactgggcgcagaacaacgcttacggcaacatctacacgatcgtttctcgtgcaatgctgaaaattctgacccgctccggctggcaaatcaaagtgattaaagaagcattcctgaccgaaaaagaacgtatctacctgctgactctgccggcaggccaggatgataaacagcagctgggcggtgatgttgtgtcccgtaccggttgccctccagtggcagttaccacttggccgctgaccctgccggtgtaataactcgagtcagataaccaggcat |
| YF1 | gtggctagttttcaatcatttgggataccaggacagctggaagtcatcaaaaaagcacttgatcacgtgcgagtcggtgtggtaattacagatcccgcacttgaagataatcctattgtctacgtaaatcaaggctttgttcaaatgaccggctacgagaccgaggaaattttaggaaagaactgtcgcttcttacaggggaaacacacagatccggcagaagtggacaacatcagaaccgctttacaaaataaagaaccggtcaccgttcagatccaaaactacaaaaaagacggaacgatgttctggaatgaattaaatattgatccaatggaaatagaggataaaacgtattttgtcggcattcagaatgatatcaccgagcaccagcagacccaggcgcgtctccaggaactgcaatccgagctcgtccacgtctccaggctgagcgccatgggcgaaatggcgtccgcgctcgcgcacgagctcaaccagccgctggcggcgatcagcaactacatgaagggctcgcggcggctgcttgccggcagcagtgatccgaacacaccgaaggtcgaaagcgccctggaccgcgccgccgagcaggcgctgcgcgccggccagatcatccggcgcctgcgcgacttcgttgcccgcggcgaatcggagaagcgggtcgagagtctctccaagctgatcgaggaggccggcgcgctcgggcttgccggcgcgcgcgagcagaacgtgcagctccgcttcagtctcgatccgggcgccgatctcgttctcgccgaccgggtgcagatccagcaggtcctggtcaacctgttccgcaacgcgctggaagcgatggctcagtcgcagcgacgcgagctcgtcgtcaccaacacccccgccgccgacgacatgatcgaggtcgaagtgtccgacaccggcagcggtttccaggacgacgtcattccgaacctgtttcagactttcttcaccaccaaggacaccggcatgggcgtgggactgtccatcagccgctcgatcatcgaagctcacggcgggcgcatgtgggccgagagcaacgcatcgggcggggcgaccttccgcttcaccctcccggcagccgacgagatga |
| fixJ | atgacgaccaagggacatatctacgtcatcgacgacgacgcggcgatgcgggattcgctgaatttcctgctggattctgccggcttcggcgtcacgctgtttgacgacgcgcaagcctttctcgacgccctgccgggtctctccttcggctgcgtcgtctccgacgtgcgcatgccgggccttgacggcatcgagctgttgaagcggatgaaggcgcagcaaagcccctttccgatcctcatcatgaccggtcacggcgacgtgccgctcgcggtcgaggcgatgaagttaggggcggtcgactttctggaaaagcctttcgaggacgaccgcctcaccgccatgatcgaatcggcgatccgccaggccgagccggccgccaagagcgaggccgtcgcgcaggatatcgccgcccgcgtcgcctcgttgagccccagggagcgccaggtcatggaagggctgatcgccggcctttccaacaagctgatcgcccgcgagtacgacatcagcccgcgcaccatcgaggtgtatcgggccaacgtcatgaccaagatgcaggccaacagcctttcggagctggttcgcctcgcgatgcgcgccggcatgctcaacgattaa |
|  |  |
